# The impact of fasting plasma glucose variability on osteoporotic fractures

**DOI:** 10.3389/fendo.2023.1187682

**Published:** 2023-06-30

**Authors:** Ri Liu, Lishu Gao, Lu Guo, Wenqi Xu, Shouling Wu, Dehu Tian

**Affiliations:** ^1^ Department of Hand Surgery, Third Hospital of Hebei Medical University, Shijiazhuang, Hebei, China; ^2^ Department of Joint Surgery, Second Hospital of Tangshan, Tangshan, Hebei, China; ^3^ Department of Endocrinology, Tangshan People’s Hospital, Tangshan, Hebei, China; ^4^ Graduate School, North China University of Science and Technology, Tangshan, Hebei, China; ^5^ Department of Cardiology, Kailuan General Hospital, Tangshan, Hebei, China

**Keywords:** osteoporotic fractures, fasting plasma glucose variability, diabetic mellitus, fasting plasma glucose, osteoporosis

## Abstract

**Purpose:**

To investigate the impact of FPG variability on osteoporotic fractures in the entire community population.

**Methods:**

All participants were from the Kailuan Study. Participants completed three consecutive surveys from 2006–2007, 2008–2009, and 2010–2011. We excluded individuals with an osteoporotic fracture in or prior to the index year and those without complete FPG records at the first 3 examinations. All participants were followed from the date of the 3rd examination to the first occurrence of an endpoint event or December 31, 2021. According to the SD of FPG levels, the included subjects were divided into three groups. A Cox proportional hazards model was performed to further analyze the effect of different FPG-SD groups on the risk of osteoporotic fractures.

**Results:**

Ultimately, the study population included 57295 participants. During a median follow-up time of 11.00 years, we documented 772 new osteoporotic fracture cases. When evaluating the FPG-SD level as a categorical variable, the HRs for osteoporotic fractures were 1.07 (95% CI: 0.89-1.29) for T2 and 1.32 (95% CI: 1.10–1.60) for T3 when compared with T1. We found that increased FPG variability was associated with a greater risk of osteoporotic fractures in people with diabetes than in those without diabetes (47% vs. 32%)

**Conclusion:**

Increased FPG variability was an independent predictor of incident osteoporotic fracture, especially in individuals older than 50 years old, nonobese individuals, diabetes patients, and individuals with positive FPG-SD variability.

## Introduction

Osteoporotic fractures are a global public health problem, with more than 8.9 million cases diagnosed each year and one osteoporotic fracture occurring every 3 seconds (1). Due to the aging of the population, the incidence of osteoporotic fractures is on the rise. Osteoporotic fractures can cause pain and severe disability. Hip and vertebral fractures can reduce patients’ life expectancy; the one-year mortality rate of long-term bedridden patients reaches 20%, and the permanent disability rate reaches 50% (2). Therefore, the early detection of osteoporotic fractures in high-risk populations is an effective prevention strategy.

Accumulating evidence has shown that diabetes increases the risk of osteoporotic fractures (3–8). In addition to fasting plasma glucose (FPG) levels, FPG variability is also associated with osteoporotic fractures. However, most relevant studies have focused on the diabetic population (9–11), and only one Korean study (12) has explored the relationship between FPG variability and osteoporotic fractures in nondiabetic people over 50 years old. There are no reports on the impact of community-wide FPG variability on osteoporotic fractures. Based on the Kailuan Study (registration number: ChiCTR-TNC-11001489), our study analyzed the impact of FPG variability on osteoporotic fractures in the entire community population to facilitate early detection and intervention, reduce the incidence of osteoporotic fractures and reduce the public health burden.

## Materials and methods

### Participants

The Kailuan Study is a large ongoing prospective cohort study. All the participants are employees and retirees of the Kailuan Group. They receive questionnaire assessments and undergo clinical examinations and laboratory tests, including FPG measurements, biennially; from June 2006 to October 2007. The occurrence of adverse events, including osteoporotic fractures, is recorded annually by the examining physician. A total of 7 follow-up visits have been completed. A detailed study design has been published elsewhere (13, 14).

All participants were from the Kailuan Study. Participants completed three consecutive surveys from 2006–2007, 2008–2009, and 2010–2011 (index year). All participants were followed from the date of the 3rd examination to the first occurrence of an endpoint event or December 31, 2021. Study included participants who 1) participated in three consecutive surveys from 2006–2007, 2008–2009 and 2010–2011; and 2) had no cognitive impairment and completed the questionnaire. We excluded individuals with an osteoporotic fracture in or prior to the index year and those without complete FPG records at the first 3 examinations. The study protocol was approved by the Ethics Committee of Kailuan General Hospital in compliance with the Declaration of Helsinki. All participants signed informed consent forms.

### Covariates assessment

Data on other related variables were collected through questionnaires (including age, sex, smoking status, alcohol drinking status, physical activity, educational level, salt intake, income level, medical history, and medication history), basic anthropometric measurements, and blood tests. Measurements of blood pressure, body mass, and height were performed according to the published literature of our group (15). All participants fasted for at least 8 h, and 5 mL of venous blood was taken on the morning of the physical examination. FPG, total cholesterol (TC), serum creatinine (Scr) and high-sensitivity C-reactive protein (hsCRP) levels were measured using a Hitachi 7600 auto-analyzer. A Japanese F-800 automatic blood cell analyzer was used to determine the hemoglobin level. The methods for the determination of the remaining biochemical parameters have been described previously (16). The estimated glomerular filtration rate (eGFR) was calculated using the Chronic Kidney Disease Epidemiology Collaboration (CKD-EPI) equation (17).

### Assessment of variability in FPG

Variability in FPG was assessed across three measures. Two indices of variability were used: standard deviation (SD): SD= $\sqrt{\frac{1}{\text{n}-1}\sum_{\text{i}-1}^{\text{n}}{\left({\text{x}}_{\text{i}}-\overset{—}{\text{x}}\right)}^{2}}$ ; and the coefficient of variation (CV): CV= (SD/mean) ×100%. A regression analysis was conducted to determine changes in FPG (from the physical examination) over time (years). The slope of this regression line represented the overall trend of FPG variability during physical examination. In this study, a slope > 0 indicated positive variation, and a slope ≤0 indicated negative variation.

### Relevant definitions and diagnostic criteria

Osteoporosis fractures were defined as low-energy or nonviolent fractures occurring without obvious external force or with the force of a fall from or below standing height. The disease diagnosis was determined by International Classification of Diseases, tenth revision (ICD-10) diagnostic codes. Osteoporotic fracture data were obtained by using the database of municipal social insurance institutions. To ensure the accuracy of osteoporotic fracture diagnosis, specialists check the basic patient information and imaging data (x-ray, computed tomography or magnetic resonance images) in their inpatient medical records system. Diabetes (18) was defined as an FPG level ≥7.0 mmol/L, a self-reported physician diagnosis, or the self-reported use of anti-diabetic medication. Hypertension (19) was defined as SBP ≥140 mmHg or DBP ≥90 mmHg, use of antihypertensive drugs, or self-reported history of physician-diagnosed hypertension. Current smoking was defined as smoking at least one cigarette per day on average in the last year. Drinking status was defined according to the consumption of more than 300 ml of liquor (alcohol concentration > 50% volume per volume) per day for at least 1 year. Physical activity was evaluated according to the frequency of physical activity during leisure time (≥ 30 min/time) and was divided into< 3 times/week and ≥ 3 times/week. Education level was defined as a primary school, middle school or college education. Salt intake was defined as ‘heavy’ (10 grams/day), ‘medium’ (6–10 grams/day), and ‘light’ (6 grams/day). Income level was defined as<3,000 RMB/month or ≥3,000 RMB/month.

### Statistical analysis

We used SAS (Version 9.4; SAS Institute, Cary, NC) for statistical analysis. For baseline descriptions, the mean ± standard deviation (SD) are used for normally distributed variables, and the median with interquartile range (25%, 75%) are used for variables with a skewed distribution. Numbers and percentages (%) are used to describe categorical variables. Normally distributed variables were compared using one-way ANOVA, while skewed variables were compared using the Kruskal−Wallis test. Categorical variables were compared using the chi-square test. According to the SD of FPG (FPG-SD) levels, the included subjects were divided into three groups: the T1 group: subjects with an FPG-SD<0.33 mmol/L; the T2 group: subjects with an FPG-SD of 0.33-0.60 mmol/L; and theT3 group: subjects with an FPG-SD≥0.60 mmol/L. Kaplan-Meier survival curves and log-rank test were used to compare osteoporotic fractures risk between groups. The incidence density of new-onset osteoporotic fractures was calculated by dividing the number of endpoints by the total person-years of follow-up (1,000 person-years). A Cox proportional hazards model was used to further analyze the effect of different FPG-SD groups on the risk of osteoporotic fractures. Model was adjusted for sex and age, smoking status, alcohol consumption status, physical activity, salt intake status, educational level and income level, mean arterial blood pressure (MAP), hemoglobin level, TC level, body mass index (BMI), hs-CRP level, eGFR, FPG level at the index year, hypoglycemic drug use, antihypertensive drug use, and lipid-lowering drug use. Restricted cubic spline models with three knots (25th, 50th, and 75th percentiles) were used to explore the patterns of associations between FPG-SD levels and the risk of osteoporotic fractures. We also performed subgroup analyses by diabetes mellitus status (including those diagnosed with diabetes at the index year and during follow-up), sex, age (<50 or ≥50 years), BMI (<28 or ≥28 kg/m^2^), and slope (≤0 or >0). Sensitivity analyses were performed to verify the robustness of the study findings. We excluded participants with drug use (including antihypertensive drugs, hypoglycemic drugs, and lipid-lowering drugs), a history of cancer, a history of stroke, and a history of atrial fibrillation (AF), and used Fine-Gray models to account for the competing risk of death. *p*< 0.05 was considered significant for 2-sided tests.

## Results

### Baseline characteristics

**Figure 1:**
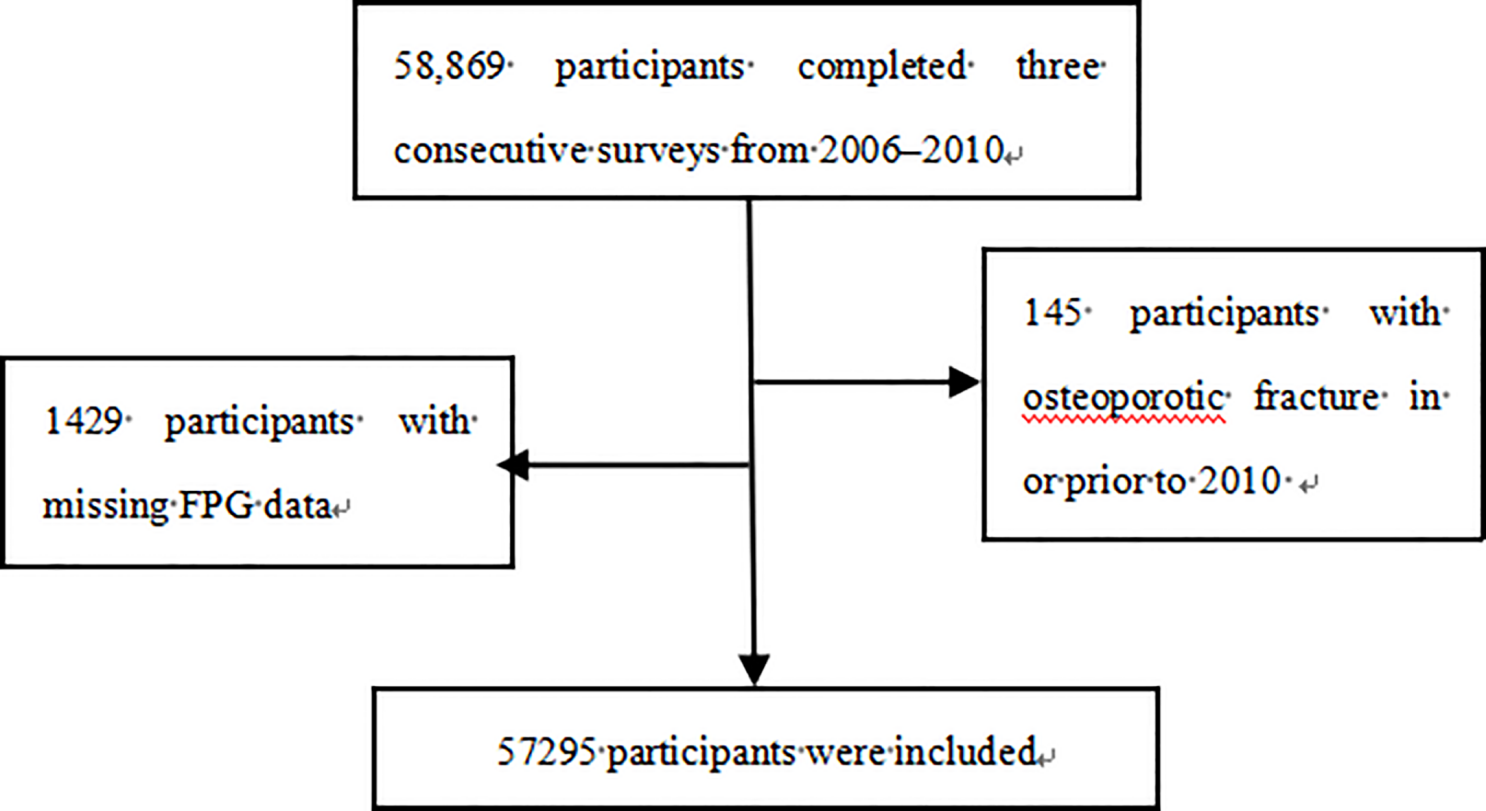
Flow chart with inclusion and exclusion criteria. FPG, fasting plasma glucose.

A total of 58,869 participants were enrolled. We excluded those with a history of osteoporotic fractures in or prior to 2010-2011 (*n*=145) and those with missing FPG data (*n*=1,429). Ultimately, the study population included 57295 participants (**Figure 1**); 76.61% were men, and the mean ± SD for age was 53.47 ± 12.15 years. The mean ± SD for FPG-SD levels was 0.61 ± 0.64 mmol/L. The sample sizes by FPG-SD level were 19,081 in the T1 group, 19,148 in the T2 group, and 19,066 in the T3 group.

Compared with the participants in T1, the participants with higher FPG-SD levels were older, had higher BMI, MAP, FPG levels, hemoglobin levels, TC levels, and hs-CRP levels, were more likely to be men, smokers and drinkers, had higher physical activity, heavy salt intake levels, and high income levels, and had higher prevalences of prior cancer, stroke, AF, hypertension, hypoglycemic drug use, antihypertensive drug use, and lipid-lowering drug use. However, they were more likely to have lower eGFRs, follow-up times and proportions of higher education levels. The comparison between groups showed significant differences (*p<* 0.05; **Table 1**). 

**Table 1:** Baseline characteristics of subjects according to the variability (SD) in FPG.

	All(n=57295)	T1(n=19081)	T2(n=19148)	T3(n=19066)	*p*
Age, years	53.47±12.15	51.74±12.12	53.23±12.26	55.45±11.78	<0.001
Male	43896(76.61)	13942(73.07)	14696(76.75)	15258(80.03)	<0.001
BMI	25.11±3.35	24.82±3.29	25.02±3.31	25.50±3.39	<0.001
MAP	99.84±12.68	98.17±12.49	99.58±12.63	101.79±12.66	<0.001
FPG(2010)	5.63±1.45	5.17±0.59	5.32±0.75	6.41±2.12	<0.001
variability (SD)	0.45(0.27-0.70)	0.21(0.15-0.27)	0.45(0.38-0.51)	0.87(0.70-1.23)	<0.001
variability (CV)	8.49(5.28-13.08)	4.15(2.82-5.31)	8.54(7.41-9.91)	15.66(13.02-20.34)	<0.001
hemoglobin	148.46±15.15	147.17±15.16	148.50±15.24	149.70±14.93	<0.001
TC	5.00±0.99	4.94±0.95	4.98±0.97	5.09±1.03	<0.001
CRP	1.20(0.63-2.80)	1.14(0.60-2.53)	1.20(0.60-2.66)	1.38(0.70-3.11)	<0.001
eGFR	89.97±18.93	91.28±18.94	90.16±18.81	88.45±18.92	<0.001
Follow-up time	11.00(10.58-11.30)	11.01(10.60-11.31)	11.00(10.59-11.29)	10.97(10.53-11.29)	<0.001
Smoking status, n(%)					
never	35322(61.65)	12110(63.47)	11756(61.40)	11456(60.09)	<0.001
past or current smoker	21973(38.35)	6971(36.53)	7392(38.60)	7610(39.91)	
Alcohol drinking, n(%)					
never	36970(64.53)	12418(65.08)	12238(63.91)	12314(64.59)	0.057
past or current drinking	20325(35.47)	6663(34.92)	6910(36.09)	6752(35.41)	
Physical activity(≥ 30 min/time), n(%)					
never or occasionally	48844(85.25)	16396(85.93)	16291(85.08)	16157(84.74)	0.004
≥ 4times/week	8451(14.75)	2685(14.07)	2857(14.92)	2909(15.26)	
Educational level, n(%)					
primary	41344(72.16)	12979(68.02)	13628(71.17)	14737(77.29)	<0.001
middle or college	15951(27.84)	6102(31.98)	5520(28.83)	4329(22.71)	
Salt, n(%)					
light or medium salt	51371(89.66)	17205(90.17)	17191(89.78)	16975(89.03)	0.001
heavy salt	5924(10.34)	1876(9.83)	1957(10.22)	2091(10.97)	
Income, n(%)					
< 3000RMB/month	49805(87.09)	17013(89.34)	16715(87.40)	16077(84.52)	<0.001
≥ 3000RMB/month	7383(12.91)	2029(10.66)	2410(12.60)	2944(15.48)	
Cancer history, n(%)	2606(4.55)	767(4.02)	867(4.53)	970(5.09)	<0.001
Stroke, n(%)	3531(6.16)	934(4.89)	1074(5.61)	1523(7.99)	<0.001
AF, n(%)	866(1.51)	246(1.29)	267(1.39)	353(1.85)	<0.001
Diabetes(all), n(%)	12798(22.34)	2011(10.54)	2711(14.16)	8076(42.36)	<0.001
antihypertensive drugs, n(%)	9137(15.95)	2429(12.73)	2924(15.27)	3784(19.85)	<0.001
hypoglycemic drugs, n(%)	2567(4.48)	163(0.85)	288(1.50)	2116(11.10)	<0.001
lipid-lowering drugs, n(%)	610(1.06)	139(0.73)	193(1.01)	278(1.46)	<0.001

Footnotes: BMI, body mass index; MAP, mean arterial blood pressure, defined as 1/3 SBP ± 2/3 DBP; FPG, fasting plasma glucose; TC, total cholesterol; CRP, C reactive protein; eGFR, estimated glomerular filtration rate; smoking status, alcohol drinking, physical activity, educational level, salt, income; stroke (including ischemic stroke, intracerebral hemorrhage and subarachnoid hemorrhage); AF, atrial fibrillation.

### Variability in FPG and osteoporotic fracture risk

**Figure 2:**
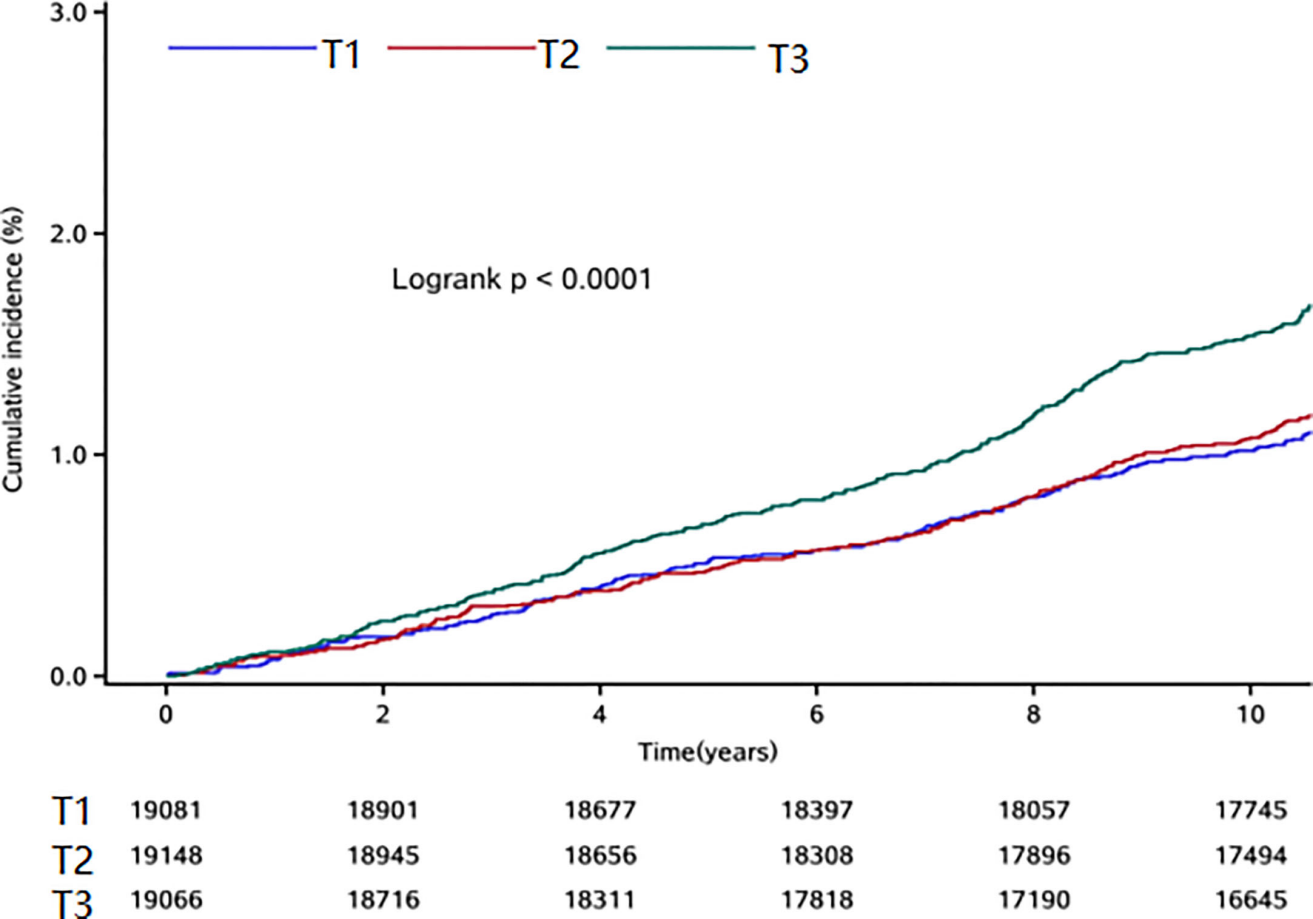
Kaplan-Meier curve for the osteoporotic fractures in T1, T2 and T3 groups.

**Figure 3:**
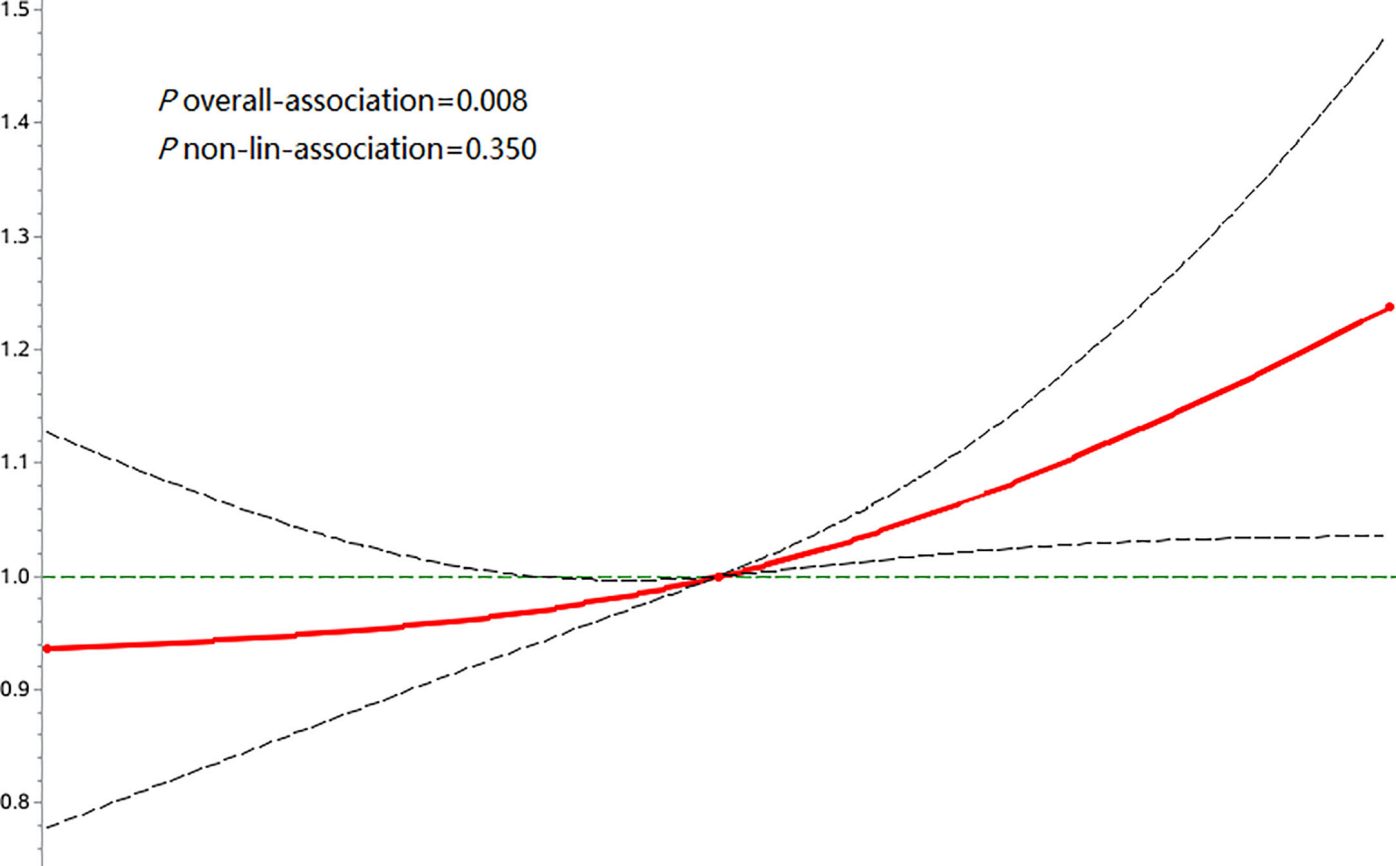
Associations of FPG-SD with risk of osteoporotic fractures using restricted cubic spline regression models.

During a median follow-up time of 11.00 years (interquartile range: 10.58-11.30), we documented 772 new osteoporotic fracture cases. There were 215 (1.13%), 237 (1.24%) and 320 (1.68%) cases of osteoporotic fractures in the T1, T2 and T3 groups, respectively. With increasing FPG-SD levels, the cumulative incidence of the endpoint tended to increase (log-rank test, p< 0.05) (**Figure 2**). The incidence densities among the three groups were 1.06, 1.18 and 1.63/1,000 person-years, respectively. When evaluating the FPG-SD level as a categorical variable, the multivariable-adjusted HRs for osteoporotic fractures were 1.07 (95% CI: 0.89-1.29) for T2 and 1.32 (95% CI: 1.10–1.60) for T3 when compared with T1 after adjusting for the following: age, sex, smoking status, alcohol consumption status, physical activity, salt intake status, educational level, income level, MAP, hemoglobin level, TC level, BMI, hs-CRP level, eGFR, FPG level at the index year, hypoglycemic drug use, antihypertensive drug use, and lipid-lowering drug use. Similar significant results were observed according to the three FPG-CV groups (**Table 2**). The restricted cubic spline models showed positive linear relationships between FPG-SD levels and the risk of incident osteoporotic fractures (*P* overall association = 0.008 and *P* nonlinear association =0.350) (**Figure 3**).

**Table 2:** Hazard ratios and 95% confidence intervals of fracture according to the different level of variability (SD and CV) of FPG.

		Subjects n	Case n(%)	Incidence rate (per 1000 person years)	Model 1	Model 2	Model 3
FPG-SD	T1	19081	215(1.13)	1.06	(ref)	(ref)	(ref)
	T2	19148	237(1.24)	1.18	1.08(0.90-1.30)	1.07(0.89-1.29)	1.07(0.89-1.29)
	T3	19066	320(1.68)	1.63	1.45(1.21-1.72)	1.43(1.20-1.70)	1.32(1.10-1.60)
	*p* trend	–	–	–	<0.001	<0.001	0.003
FPG-CV	T1	19079	221(1.16)	1.09	(ref)	(ref)	(ref)
	T2	19139	240(1.25)	1.19	1.06(0.88-1.27)	1.06(0.88-1.27)	1.05(0.87-1.26)
	T3	19077	311(1.63)	1.58	1.36(1.14-1.61)	1.34(1.13-1.60)	1.25(1.05-1.50)
	*p* trend	–	–	–	<0.001	<0.001	0.013

P for trend, P value for trend across the different level of FPG-SD and FPG-CV; Per SD, hazard ratio for per standard deviation change in FPG-SD and FPG-CV. Model 1 was adjusted for gender, age; Model 2 was further adjusted for smoking status, alcohol drinking, physical activity, educational leve, salt, income; Model 3 was further adjusted for MAP (mmHg), hemoglobin (g/L), TC (mmol/L), BMI (kg/m^2^), CRP (mg/L), eGFR (ml/min/1.73m^2^), FPG in 2010, hypoglycemic drug use, antihypertensive drug use, and lipid-lowering drug use.

### Subgroup analysis and sensitivity analysis

To investigate the effect of FPG variability on osteoporotic fractures in participants with and without diabetes, we divided the population into groups with or without diabetes. The diabetes group included participants diagnosed with diabetes at baseline and during follow-up, and the HR and 95% CI of the risk of osteoporotic fractures in the T2 and T3 groups were 1.62(1.12-2.34) and 1.47(1.00-2.15), respectively. In the nondiabetic population, the HRs and 95% CIs of osteoporotic fractures in the T2 and T3 groups were 1.12(0.91-1.39) and 1.32(1.08-1.61), respectively, compared with the T1 group. In the sex stratification, after adjusting for risk factors, the HRs and 95% CIs for osteoporotic fracture risk were 1.27(1.01-1.59) and 1.42(1.01-1.99) for the male and female T3 groups compared with the T1 group. Among patients older than 50 years, the HR and 95% CI for the risk of osteoporotic fractures was 1.46(95% CI: 1.17-1.20) in the T3 group compared with the T1 group, and FPG variability was nonsignificantly associated with osteoporotic fractures in the group aged< 50 years. In BMI stratification, the HR and 95% CI of osteoporotic fracture risk in the nonobese (BMI<28kg/m^2^) T3 group was 1.33(1.08-1.64), and FPG variability in the obese group (BMI≥28 kg/m^2^) was 1.30(0.84-2.01) (*p >*0.05). When stratified according to the slope, the HR value and 95% CI of the FPG-SD positive change group (T3) for osteoporotic fracture risk was 1.37(1.08-1.74), while the negative change group was 1.25(0.92-1.70) (*p >*0.05). We found no significant interaction of the above factors stratified by FPG variability in relation to the risk of osteoporotic fractures (*p* for interaction >0.05 for all) (**Table 3**). In the sensitivity analysis excluding individuals with a history of antihypertensive drug, hypoglycemic drug, or lipid-lowering drug use, a history of cancer, a history of stroke, and a history of atrial fibrillation, increasing FPG-SD levels still predicted osteoporotic fracture risk. In the competing risk analyses, the results from the Fine-Gray model were similar to the main results (**Table 4**).

**Table 3:** Subgroup analysis for hazard ratios and 95% confidence intervals of fracture according to the quartiles of variability (SD) of FPG.

		Subjects N	Case,n(%)	T1	T2	T3	*P* for interaction
DM or not	DM	12798	187(1.46)	(ref)	1.62(1.12-2.34)	1.47(1.00-2.15)	0.532
	Without-DM	44497	585(1.31)	(ref)	1.12(0.91-1.39)	1.32(1.08-1.61)	
gender	male	43896	533(1.21)	(ref)	1.06(0.85-1.32)	1.27(1.01-1.59)	0.172
	female	13399	239(1.78)	(ref)	1.06(0.76-1.49)	1.42(1.01-1.99)	
Age (years)	<50	22035	197(0.89)	(ref)	0.94(0.67-1.32)	1.03(0.71-1.48)	0.387
	≥50	35260	575(1.63)	(ref)	1.14(0.91-1.42)	1.46(1.17-1.2)	
BMI(kg/m^2^)	<28	46859	620(1.32)	(ref)	1.07(0.87-1.31)	1.33(1.08-1.64)	0.794
	≥28	10436	152(1.46)	(ref)	1.09(0.70-1.69)	1.30(0.84-2.01)	
slope	Slope≤0	21725	261(1.20)	(ref)	0.91(0.66-1.25)	1.25(0.92-1.70)	0.698
	Slope>0	35570	511(1.44)	(ref)	1.15(0.91-1.44)	1.37(1.08-1.74)	

Model was adjusted for gender(not adjusted in subgroup analysis by gender), age(not adjusted in subgroup analysis by age), smoking status, alcohol drinking, physical activity, educational level, salt, income, MAP (mmHg), hemoglobin (g/L), TC (mmol/L), BMI (kg/m^2^) (not adjusted in subgroup analysis by BMI), CRP(mg/L), eGFR(ml/min/1.73m^2^), FPG in 2010, hypoglycemic drug use, antihypertensive drug use, and lipid-lowering drug use.

**Table 4:** Sensitive analyses for hazard ratios values and 95% Confidence Intervals (CI).

	T1	T2	T3
Sensitive analyses 1	(ref)	1.06(0.86-1.29)	1.27(1.03-1.57)
Sensitive analyses 2	(ref)	1.10(0.91-1.33)	1.34(1.11-1.63)
Sensitive analyses 3	(ref)	1.09(0.89-1.32)	1.34(1.10-1.63)
Sensitive analyses 4	(ref)	1.10(0.91-1.32)	1.35(1.12-1.64)
Sensitive analyses 5	(ref)	1.08(1.00-1.16)	1.23(1.15-1.33)

Model was adjusted for gender, age, smoking status, alcohol drinking, physical activity, educational level, salt, income; MAP (mmHg), hemoglobin (g/L), TC (mmol/L), BMI (kg/m^2^), CRP(mg/L), eGFR(ml/min/1.73m^2^), FPG in 2010, hypoglycemic drug use, antihypertensive drug use, and lipid-lowering drug use. Sensitive analyses 1 was for the participants without using drugs (n=10675). Sensitive analyses 2 was for the participants without cancer (n=2604). Sensitive analyses 3 was for the participants without stroke (n=3531). Sensitive analyses 4 was for the participants without AF (n=866). Sensitive analyses 5 was for the competing risk of death (n=4775).

## Discussion

Our study provides the first report on the effect of FPG variability on osteoporotic fracture risk in the entire community population. We found that increased FPG variability was an independent predictor of incident osteoporotic fracture, especially in individuals older than 50 years old, nonobese individuals, diabetes patients, and individuals with positive FPG-SD variability.

An important finding of this study is that high FPG variability was a risk factor for osteoporotic fractures, with a 32% increased risk of osteoporotic fractures in the T3 group compared with the T1 group, even after adjustment for possible confounders. In addition, although there was no statistically significant increase in the risk of osteoporotic fracture in the T2 group, the *p* trend was<0.001, and the restricted cubic spline regression model showed *p*-overall=0.008 and *p*-nonlinear =0.350, suggesting a linear relationship between FPG variability and osteoporotic fracture risk. Studies (20, 21) have found that frailty and malnutrition are risk factors for osteoporotic fractures. Although our study did not directly measure the participants’ nutritional levels, we adjusted for hemoglobin, which is a variable that indirectly represents the nutritional level (22), and high FPG variability is still a risk factor for osteoporotic fractures. In addition, increased FPG variability remained independently associated with osteoporotic fractures after excluding subjects with a history of drug use, cancer, stroke, and atrial fibrillation and after accounting for competing risks of death. This supports the robustness of our results.

There was only one Korean study (12) that addressed the association between FPG variability and osteoporotic fractures in a nondiabetic population, reporting an 11% increase in the risk of osteoporotic fractures in the fourth quartile of FPG variability compared with the first quartile. Our stratified results showed a 32% increased risk of osteoporotic fractures in the third tertile of FPG variability compared with the first tertile in the nondiabetic population. Both the Korean study and our studies support the conclusion that high FPG variability is a risk factor for osteoporotic fractures in a nondiabetic population. At the same time, we found that increased FPG variability was associated with a greater risk of osteoporotic fractures in people with diabetes than in those without diabetes (47% vs. 32%). A study (11) of a diabetic population from Hong Kong, China, showed a 48% increased risk of hip fractures in the fourth quartile of HbA1c variability compared with the first quartile after adjustment for associated risk factors. A study (9) on FPG variability and hip fractures in people with diabetes in Taiwan, China, showed that the risk of hip fractures in the fourth quartile of FPG variability was 27% higher than that in the first quartile. Both of these studies of diabetic populations were similar to our results for a diabetic population.

Although we did not find an interaction among age, sex, and BMI in the overall population, we performed a subgroup analysis based on previous findings that age, sex, and BMI may have some effect on osteoporotic fracture risk. In our study, compared with the first tertile, the third tertile of FPG variability increased the risk of osteoporotic fractures by 42% in women and only 27% in men. Studies (23–25) in the United States, Sweden, and the United Kingdom suggest that women have a higher risk of osteoporotic fractures than men in the general population, which is consistent with our results. In terms of age, a study (23) suggested that the incidence of osteoporotic fractures increased after the age of 50 years, which was similar to our conclusion in the age group analysis; that is, high FPG variability increased the risk of osteoporotic fractures only in people older than 50 years, and there was no significant difference in people younger than 50 years. Although the effect of BMI on osteoporotic fractures is currently inconclusive, our study supports that increased FPG variability is a risk factor for osteoporotic fractures in the nonobese population but not in the obese population.

Our results not only confirm the results of previous studies but also further expand the knowledge in this field. When stratified according to the slope, we found that increased FPG variability was associated with higher osteoporotic fracture risk only in the positive variant population, whereas it did not reach statistical significance in the negative variant population. A study (26) on the effect of HbA1c changes on fracture risk in a diabetic population over two years showed that a 1% increase in longitudinal HbA1c levels would lead to an 8% increase in fracture risk, which also supported the conclusion that the risk of fracture increased in people with elevated blood glucose levels. This finding suggests that we should pay attention not only to FPG variability but also to the forward variation in FPG because forward variation may represent deterioration due to glucose metabolism disorder. Early intervention to prevent further deterioration of glucose metabolism may reduce the incidence of complications, including osteoporotic fractures.

The mechanisms underlying the associations between FPG variability and osteoporotic fractures are likely to be driven by several explanations. First, FPG variability is related to oxidative stress (27). Transient hyperglycemia has been shown to induce long-lasting activating epigenetic changes in the promoter of the nuclear factor kappa-B subunit p65 in aortic endothelial cells, which causes increased p65 gene expression, both *in vitro* and in nondiabetic mice (28). Human studies have also shown that fluctuations in glucose levels are worse than high mean glucose levels in terms of oxidative stress and endothelial dysfunction (29). Oxidative stress is associated with an imbalance in osteoblast and osteoclast activity, which leads to increased turnover in bone remodeling and bone loss (30). In addition, elevated proinflammatory and proabsorptive cytokines caused by changes in blood glucose levels can lead to bone loss (31). Since damage to the microvascular system is associated with the deterioration of bone microstructure, high FPG variability may lead to impaired bone quality, thereby increasing the risk of fractures (32).

### Strengths and limitations

The present study had several strengths. First, we based our study on the Kailuan cohort, which included a large population and long-term follow-up information. Second, the analysis of FPG variability was performed using two different variability measures (FPG-SD, FPG-CV) and extensive subgroup and sensitivity analyses to ensure the robustness of the findings. Third, we divided the study population into groups with or without diabetes and investigated the effect of different glycemic variability on osteoporotic fractures in the entire population and diabetic and nondiabetic populations. However, several limitations should also be noted. First, this was an observational cohort study, so pathophysiological mechanisms cannot be inferred but only speculated. Second, we cannot exclude the possibility of residual or unmeasured confounding, such as antiosteoporosis drug use, parathyroid hormone level, and bone mineral density, given the observational design of the present analysis. Finally, the participants were all from the Kailuan community and they were not nationally representative of the Chinese population. Compared with the general adult population, the proportion of men is higher in our study. These factors may limit the generalization of our findings.

## Conclusion

Our study is the first to demonstrate that increased FPG variability is a risk factor for incident osteoporotic fractures in a community-based population.

## Data availability statement

The original contributions presented in the study are included in the article/**Supplementary Material**. Further inquiries can be directed to the corresponding author.

## Ethics statement

The studies involving human participants were reviewed and approved by The Ethics Committee of the Kailuan General Hospital. The patients/participants provided their written informed consent to participate in this study.

## Author contributions

All authors read and approved the final version of the manuscript. RL and LSG wrote the manuscript. LG and WX researched the data. RL and SW contributed to the study design and discussion. DT contributed to the discussion and reviewed/edited the manuscript.
