# Supplementary material for: The impact of fasting plasma glucose variability on osteoporotic fractures
Source: Front Endocrinol (Lausanne). 2023 Jun 30;14:1187682. doi: 10.3389/fendo.2023.1187682 (PMC10348823; doi:10.3389/fendo.2023.1187682)
Supplement: Supplementary file 1 [file Table_1.docx]

Supplementary table 1. Distribution of fracture location

| Fracture location | Number of cases, n |
| --- | --- |
| Hip | 234 |
| Vertebral, rib, clavicular, scapular, and sternal | 172 |
| Fractures of upper extremities | 148 |
| Fractures of the lower extremities | 192 |
| Other fractures | 26 |
| Total | 772 |

Fractures of Hip include fractures of the fracture of neck of femur, pertrochanteric fracture, and subtrochanteric fracture. Fractures of upper extremities include fractures of the humerus and forearm. Fractures of the lower extremities include fractures of the pelvis, tibia, fibula, and other femoral fractures. Other fractures (site of fracture was unclear but diagnosis of fracture).
